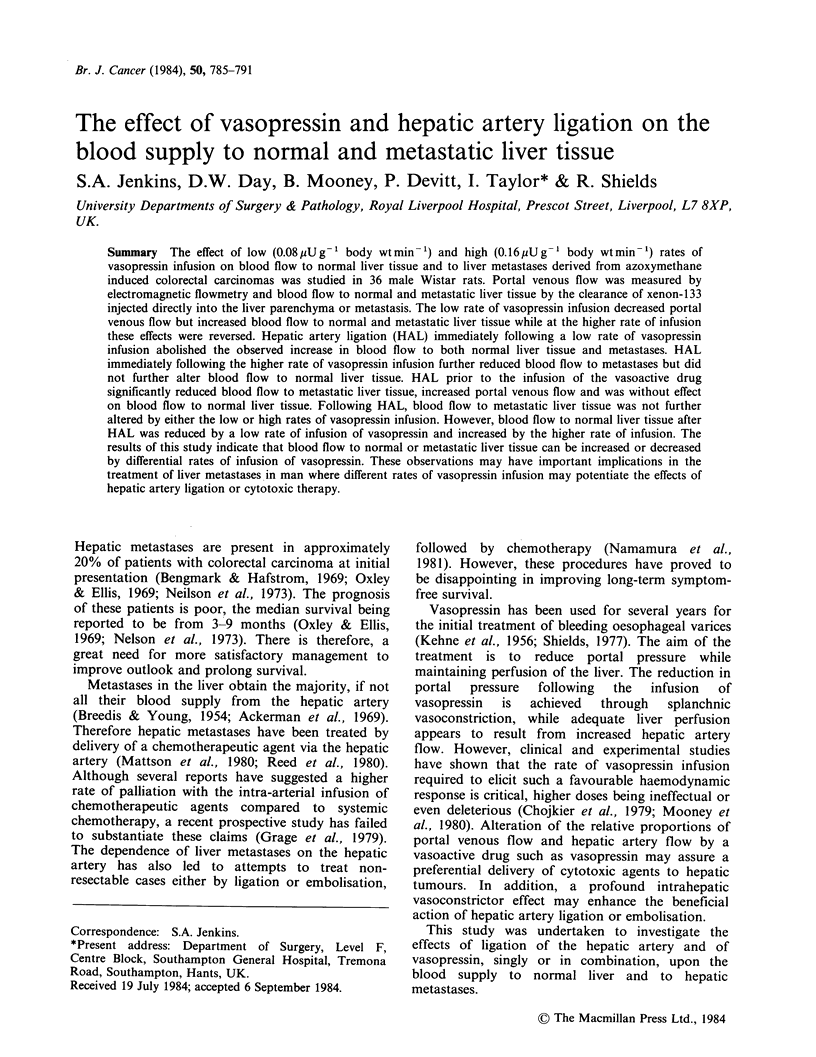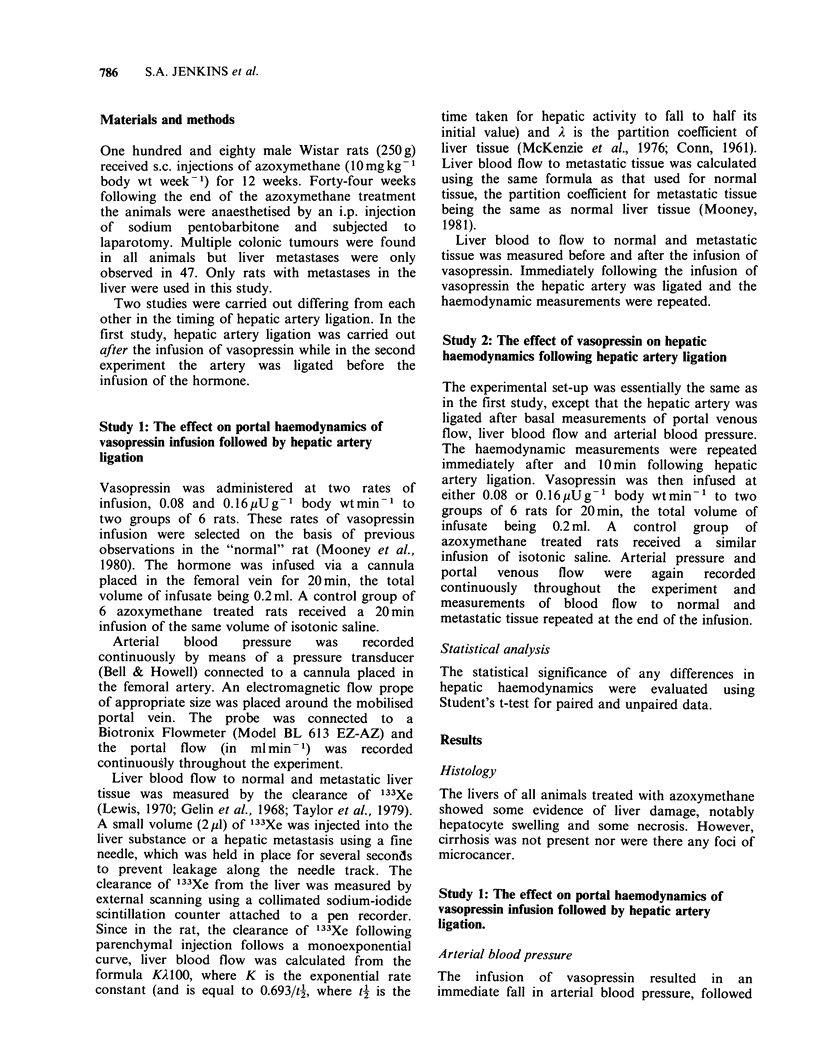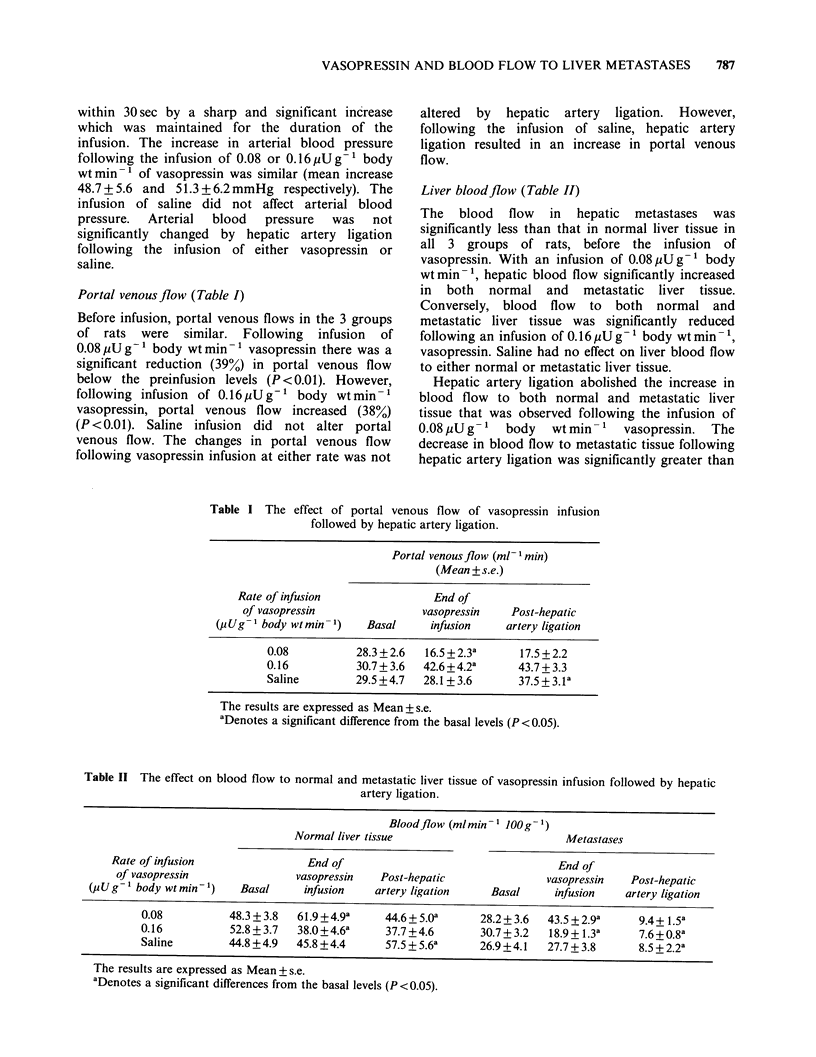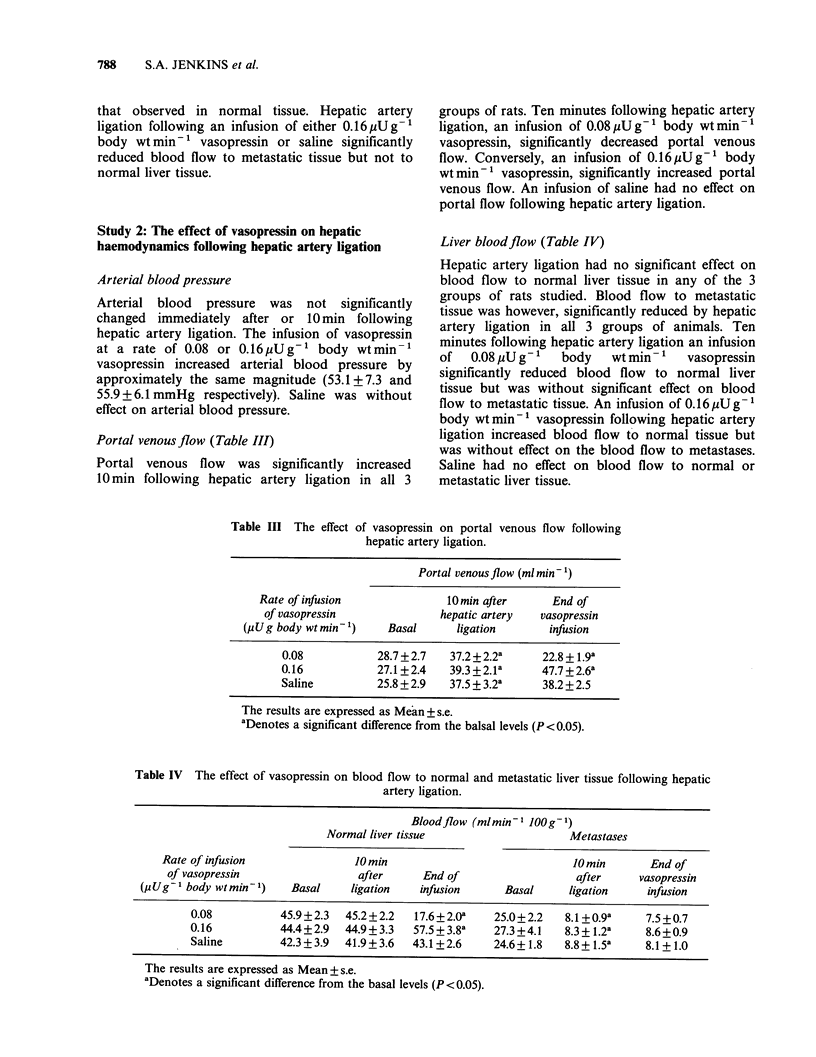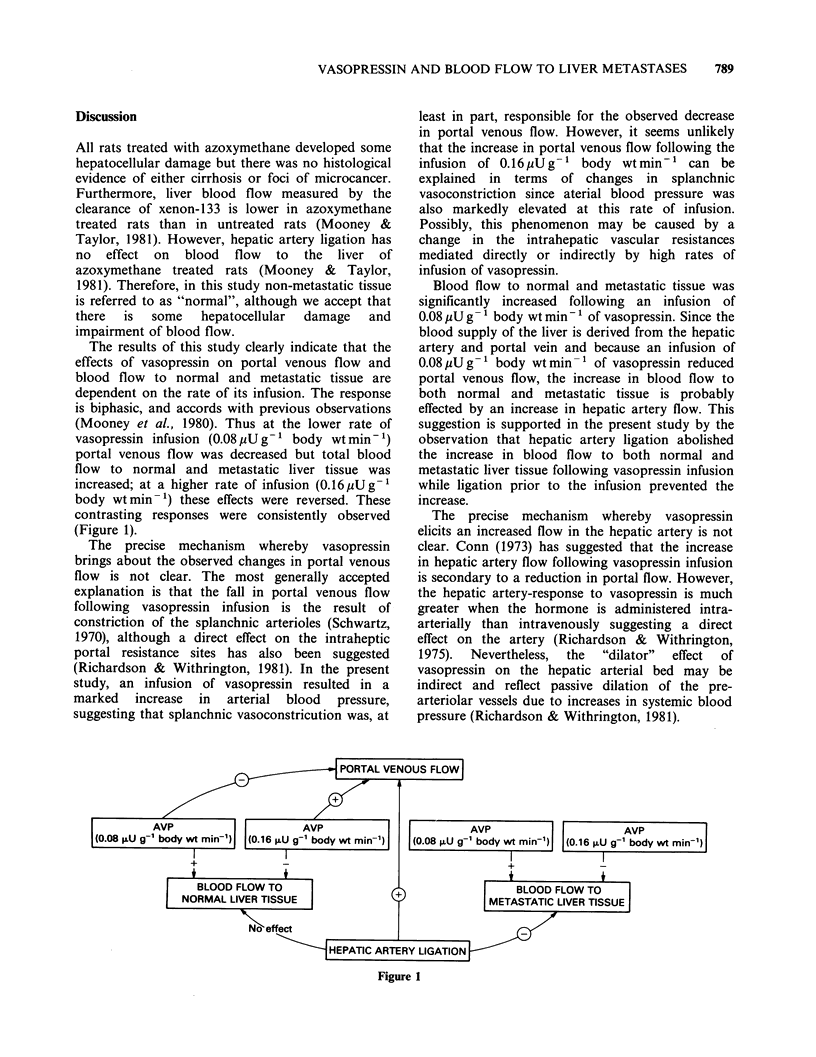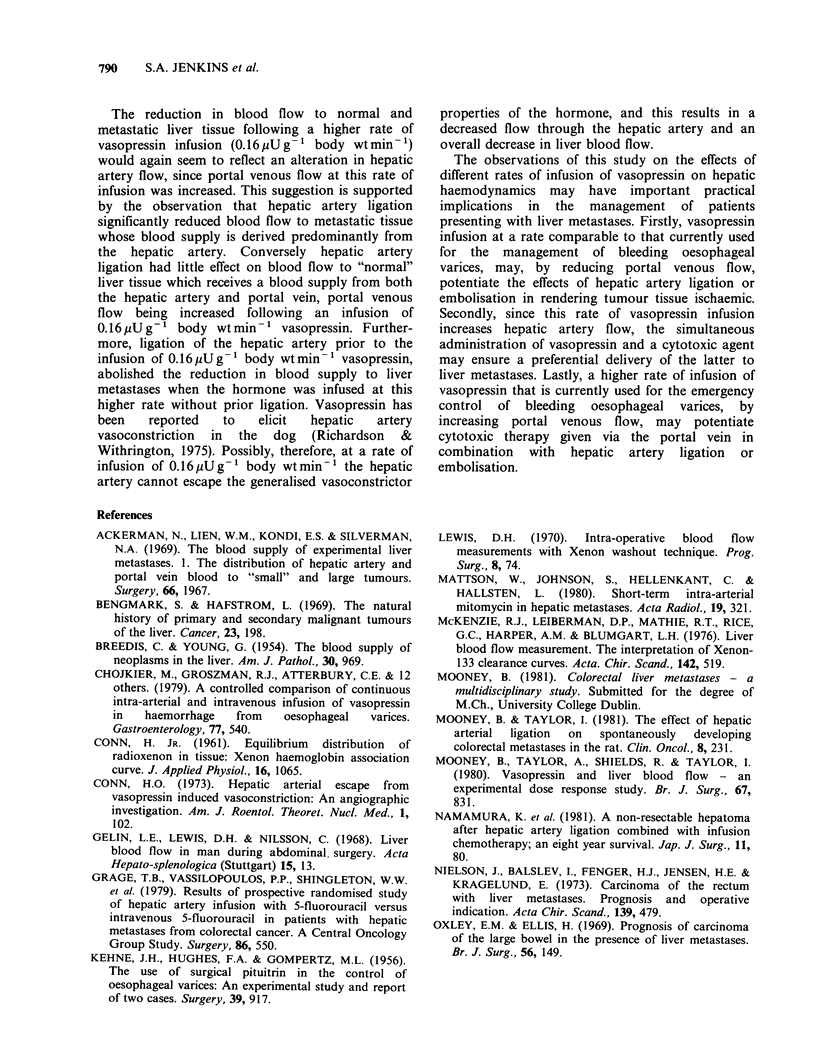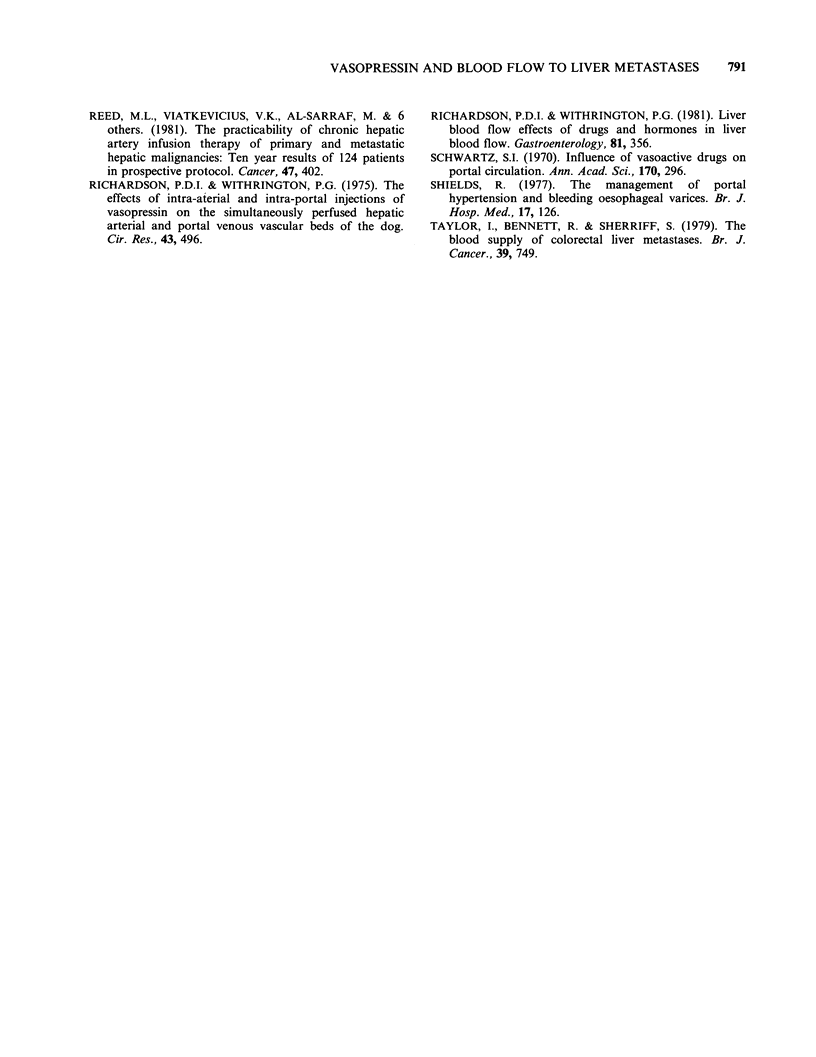# The effect of vasopressin and hepatic artery ligation on the blood supply to normal and metastatic liver tissue.

**DOI:** 10.1038/bjc.1984.257

**Published:** 1984-12

**Authors:** S. A. Jenkins, D. W. Day, B. Mooney, P. Devitt, I. Taylor, R. Shields

## Abstract

The effect of low (0.08 microU g-1 body wt min-1) and high (0.16 microU g-1 body wt min-1) rates of vasopressin infusion on blood flow to normal liver tissue and to liver metastases derived from azoxymethane induced colorectal carcinomas was studied in 36 male Wistar rats. Portal venous flow was measured by electromagnetic flowmetry and blood flow to normal and metastatic liver tissue by the clearance of xenon-133 injected directly into the liver parenchyma or metastasis. The low rate of vasopressin infusion decreased portal venous flow but increased blood flow to normal and metastatic liver tissue while at the higher rate of infusion these effects were reversed. Hepatic artery ligation (HAL) immediately following a low rate of vasopressin infusion abolished the observed increase in blood flow to both normal liver tissue and metastases. HAL immediately following the higher rate of vasopressin infusion further reduced blood flow to metastases but did not further alter blood flow to normal liver tissue. HAL prior to the infusion of the vasoactive drug significantly reduced blood flow to metastatic liver tissue, increased portal venous flow and was without effect on blood flow to normal liver tissue. Following HAL, blood flow to metastatic liver tissue was not further altered by either the low or high rates of vasopressin infusion. However, blood flow to normal liver tissue after HAL was reduced by a low rate of infusion of vasopressin and increased by the higher rate of infusion. The results of this study indicate that blood flow to normal or metastatic liver tissue can be increased or decreased by differential rates of infusion of vasopressin. These observations may have important implications in the treatment of liver metastases in man where different rates of vasopressin infusion may potentiate the effects of hepatic artery ligation or cytotoxic therapy.


*Br. J. Cancer* (1984), **50**, 785–791

### The effect of vasopressin and hepatic artery ligation on the blood supply to normal and metastatic liver tissue

S.A. Jenkins, D.W. Day, B. Mooney, P. Devitt, I. Taylor* & R. Shields

*University Departments of Surgery & Pathology, Royal Liverpool Hospital, Prescot Street, Liverpool, L7 8XP, UK.*

**Summary** The effect of low ($0.08\mu U g^{-1}$ body wt $min^{-1}$) and high ($0.16\mu U g^{-1}$ body wt $min^{-1}$) rates of vasopressin infusion on blood flow to normal liver tissue and to liver metastases derived from azoxymethane induced colorectal carcinomas was studied in 36 male Wistar rats. Portal venous flow was measured by electromagnetic flowmetry and blood flow to normal and metastatic liver tissue by the clearance of xenon-133 injected directly into the liver parenchyma or metastasis. The low rate of vasopressin infusion decreased portal venous flow but increased blood flow to normal and metastatic liver tissue while at the higher rate of infusion these effects were reversed. Hepatic artery ligation (HAL) immediately following a low rate of vasopressin infusion abolished the observed increase in blood flow to both normal liver tissue and metastases. HAL immediately following the higher rate of vasopressin infusion further reduced blood flow to metastases but did not further alter blood flow to normal liver tissue. HAL prior to the infusion of the vasoactive drug significantly reduced blood flow to metastatic liver tissue, increased portal venous flow and was without effect on blood flow to normal liver tissue. Following HAL, blood flow to metastatic liver tissue was not further altered by either the low or high rates of vasopressin infusion. However, blood flow to normal liver tissue after HAL was reduced by a low rate of infusion of vasopressin and increased by the higher rate of infusion. The results of this study indicate that blood flow to normal or metastatic liver tissue can be increased or decreased by differential rates of infusion of vasopressin. These observations may have important implications in the treatment of liver metastases in man where different rates of vasopressin infusion may potentiate the effects of hepatic artery ligation or cytotoxic therapy.

Hepatic metastases are present in approximately 20% of patients with colorectal carcinoma at initial presentation (Bengmark & Hafstrom, 1969; Oxley & Ellis, 1969; Neilson *et al.*, 1973). The prognosis of these patients is poor, the median survival being reported to be from 3–9 months (Oxley & Ellis, 1969; Nelson *et al.*, 1973). There is therefore, a great need for more satisfactory management to improve outlook and prolong survival.

Metastases in the liver obtain the majority, if not all their blood supply from the hepatic artery (Breedis & Young, 1954; Ackerman *et al.*, 1969). Therefore hepatic metastases have been treated by delivery of a chemotherapeutic agent via the hepatic artery (Mattson *et al.*, 1980; Reed *et al.*, 1980). Although several reports have suggested a higher rate of palliation with the intra-arterial infusion of chemotherapeutic agents compared to systemic chemotherapy, a recent prospective study has failed to substantiate these claims (Grage *et al.*, 1979). The dependence of liver metastases on the hepatic artery has also led to attempts to treat non-resectable cases either by ligation or embolisation, followed by chemotherapy (Namamura *et al.*, 1981). However, these procedures have proved to be disappointing in improving long-term symptom-free survival.

Vasopressin has been used for several years for the initial treatment of bleeding oesophageal varices (Kehne *et al.*, 1956; Shields, 1977). The aim of the treatment is to reduce portal pressure while maintaining perfusion of the liver. The reduction in portal pressure following the infusion of vasopressin is achieved through splanchnic vasoconstriction, while adequate liver perfusion appears to result from increased hepatic artery flow. However, clinical and experimental studies have shown that the rate of vasopressin infusion required to elicit such a favourable haemodynamic response is critical, higher doses being ineffectual or even deleterious (Chojkier *et al.*, 1979; Mooney *et al.*, 1980). Alteration of the relative proportions of portal venous flow and hepatic artery flow by a vasoactive drug such as vasopressin may assure a preferential delivery of cytotoxic agents to hepatic tumours. In addition, a profound intrahepatic vasoconstrictor effect may enhance the beneficial action of hepatic artery ligation or embolisation.

This study was undertaken to investigate the effects of ligation of the hepatic artery and of vasopressin, singly or in combination, upon the blood supply to normal liver and to hepatic metastases.

Correspondence: S.A. Jenkins.
*Present address: Department of Surgery, Level F, Centre Block, Southampton General Hospital, Tremona Road, Southampton, Hants, UK.
Received 19 July 1984; accepted 6 September 1984.

© The Macmillan Press Ltd., 1984

### Materials and methods

One hundred and eighty male Wistar rats (250 g) received s.c. injections of azoxymethane (10 mg $kg^{-1}$ body wt $week^{-1}$) for 12 weeks. Forty-four weeks following the end of the azoxymethane treatment the animals were anaesthetised by an i.p. injection of sodium pentobarbitone and subjected to laparotomy. Multiple colonic tumours were found in all animals but liver metastases were only observed in 47. Only rats with metastases in the liver were used in this study.

Two studies were carried out differing from each other in the timing of hepatic artery ligation. In the first study, hepatic artery ligation was carried out *after* the infusion of vasopressin while in the second experiment the artery was ligated before the infusion of the hormone.

### Study 1: The effect on portal haemodynamics of vasopressin infusion followed by hepatic artery ligation

Vasopressin was administered at two rates of infusion, 0.08 and 0.16 $\mu U g^{-1}$ body wt $min^{-1}$ to two groups of 6 rats. These rates of vasopressin infusion were selected on the basis of previous observations in the "normal" rat (Mooney *et al.*, 1980). The hormone was infused via a cannula placed in the femoral vein for 20 min, the total volume of infusate being 0.2 ml. A control group of 6 azoxymethane treated rats received a 20 min infusion of the same volume of isotonic saline.

Arterial blood pressure was recorded continuously by means of a pressure transducer (Bell & Howell) connected to a cannula placed in the femoral artery. An electromagnetic flow prope of appropriate size was placed around the mobilised portal vein. The probe was connected to a Biotronix Flowmeter (Model BL 613 EZ-AZ) and the portal flow (in ml $min^{-1}$) was recorded continuously throughout the experiment.

Liver blood flow to normal and metastatic liver tissue was measured by the clearance of $^{133}Xe$ (Lewis, 1970; Gelin *et al.*, 1968; Taylor *et al.*, 1979). A small volume (2 $\mu$l) of $^{133}Xe$ was injected into the liver substance or a hepatic metastasis using a fine needle, which was held in place for several seconds to prevent leakage along the needle track. The clearance of $^{133}Xe$ from the liver was measured by external scanning using a collimated sodium-iodide scintillation counter attached to a pen recorder. Since in the rat, the clearance of $^{133}Xe$ following parenchymal injection follows a monoexponential curve, liver blood flow was calculated from the formula $K\lambda 100$, where $K$ is the exponential rate constant (and is equal to $0.693/t\frac{1}{2}$, where $t\frac{1}{2}$ is the time taken for hepatic activity to fall to half its initial value) and $\lambda$ is the partition coefficient of liver tissue (McKenzie *et al.*, 1976; Conn, 1961). Liver blood flow to metastatic tissue was calculated using the same formula as that used for normal tissue, the partition coefficient for metastatic tissue being the same as normal liver tissue (Mooney, 1981).

Liver blood to flow to normal and metastatic tissue was measured before and after the infusion of vasopressin. Immediately following the infusion of vasopressin the hepatic artery was ligated and the haemodynamic measurements were repeated.

### Study 2: The effect of vasopressin on hepatic haemodynamics following hepatic artery ligation

The experimental set-up was essentially the same as in the first study, except that the hepatic artery was ligated after basal measurements of portal venous flow, liver blood flow and arterial blood pressure. The haemodynamic measurements were repeated immediately after and 10 min following hepatic artery ligation. Vasopressin was then infused at either 0.08 or 0.16 $\mu U g^{-1}$ body wt $min^{-1}$ to two groups of 6 rats for 20 min, the total volume of infusate being 0.2 ml. A control group of azoxymethane treated rats received a similar infusion of isotonic saline. Arterial pressure and portal venous flow were again recorded continuously throughout the experiment and measurements of blood flow to normal and metastatic tissue repeated at the end of the infusion.

*Statistical analysis*

The statistical significance of any differences in hepatic haemodynamics were evaluated using Student's t-test for paired and unpaired data.

### Results

*Histology*

The livers of all animals treated with azoxymethane showed some evidence of liver damage, notably hepatocyte swelling and some necrosis. However, cirrhosis was not present nor were there any foci of microcancer.

### Study 1: The effect on portal haemodynamics of vasopressin infusion followed by hepatic artery ligation.

*Arterial blood pressure*

The infusion of vasopressin resulted in an immediate fall in arterial blood pressure, followed within 30 sec by a sharp and significant increase which was maintained for the duration of the infusion. The increase in arterial blood pressure following the infusion of 0.08 or $0.16\,\mu U\,g^{-1}$ body wt $min^{-1}$ of vasopressin was similar (mean increase $48.7 \pm 5.6$ and $51.3 \pm 6.2$ mmHg respectively). The infusion of saline did not affect arterial blood pressure. Arterial blood pressure was not significantly changed by hepatic artery ligation following the infusion of either vasopressin or saline.

*Portal venous flow (Table I)*

Before infusion, portal venous flows in the 3 groups of rats were similar. Following infusion of $0.08\,\mu U\,g^{-1}$ body wt $min^{-1}$ vasopressin there was a significant reduction (39%) in portal venous flow below the preinfusion levels ($P < 0.01$). However, following infusion of $0.16\,\mu U\,g^{-1}$ body wt $min^{-1}$ vasopressin, portal venous flow increased (38%) ($P < 0.01$). Saline infusion did not alter portal venous flow. The changes in portal venous flow following vasopressin infusion at either rate was not altered by hepatic artery ligation. However, following the infusion of saline, hepatic artery ligation resulted in an increase in portal venous flow.

*Liver blood flow (Table II)*

The blood flow in hepatic metastases was significantly less than that in normal liver tissue in all 3 groups of rats, before the infusion of vasopressin. With an infusion of $0.08\,\mu U\,g^{-1}$ body wt $min^{-1}$, hepatic blood flow significantly increased in both normal and metastatic liver tissue. Conversely, blood flow to both normal and metastatic liver tissue was significantly reduced following an infusion of $0.16\,\mu U\,g^{-1}$ body wt $min^{-1}$, vasopressin. Saline had no effect on liver blood flow to either normal or metastatic liver tissue.

Hepatic artery ligation abolished the increase in blood flow to both normal and metastatic liver tissue that was observed following the infusion of $0.08\,\mu U\,g^{-1}$ body wt $min^{-1}$ vasopressin. The decrease in blood flow to metastatic tissue following hepatic artery ligation was significantly greater than

**Table I** The effect of portal venous flow of vasopressin infusion followed by hepatic artery ligation.

| Rate of infusion of vasopressin ($\mu U g^{-1}$ body wt $min^{-1}$) | Portal venous flow ($ml^{-1}$ min) (Mean ± s.e.) Basal | End of vasopressin infusion | Post-hepatic artery ligation |
|---|---|---|---|
| 0.08 | 28.3 ± 2.6 | 16.5 ± 2.3[a] | 17.5 ± 2.2 |
| 0.16 | 30.7 ± 3.6 | 42.6 ± 4.2[a] | 43.7 ± 3.3 |
| Saline | 29.5 ± 4.7 | 28.1 ± 3.6 | 37.5 ± 3.1[a] |

The results are expressed as Mean ± s.e.
[a]Denotes a significant difference from the basal levels ($P < 0.05$).

**Table II** The effect on blood flow to normal and metastatic liver tissue of vasopressin infusion followed by hepatic artery ligation.

| | Blood flow ($ml\,min^{-1}$ $100\,g^{-1}$) Normal liver tissue | | | Metastases | | |
|---|---|---|---|---|---|---|
| Rate of infusion of vasopressin ($\mu U\,g^{-1}$ body wt $min^{-1}$) | Basal | End of vasopressin infusion | Post-hepatic artery ligation | Basal | End of vasopressin infusion | Post-hepatic artery ligation |
| 0.08 | 48.3 ± 3.8 | 61.9 ± 4.9[a] | 44.6 ± 5.0[a] | 28.2 ± 3.6 | 43.5 ± 2.9[a] | 9.4 ± 1.5[a] |
| 0.16 | 52.8 ± 3.7 | 38.0 ± 4.6[a] | 37.7 ± 4.6 | 30.7 ± 3.2 | 18.9 ± 1.3[a] | 7.6 ± 0.8[a] |
| Saline | 44.8 ± 4.9 | 45.8 ± 4.4 | 57.5 ± 5.6[a] | 26.9 ± 4.1 | 27.7 ± 3.8 | 8.5 ± 2.2[a] |

The results are expressed as Mean ± s.e.
[a]Denotes a significant differences from the basal levels ($P < 0.05$).

that observed in normal tissue. Hepatic artery ligation following an infusion of either 0.16 $\mu U g^{-1}$ body wt $min^{-1}$ vasopressin or saline significantly reduced blood flow to metastatic tissue but not to normal liver tissue.

**Study 2: The effect of vasopressin on hepatic haemodynamics following hepatic artery ligation**

*Arterial blood pressure*

Arterial blood pressure was not significantly changed immediately after or 10 min following hepatic artery ligation. The infusion of vasopressin at a rate of 0.08 or 0.16 $\mu U g^{-1}$ body wt $min^{-1}$ vasopressin increased arterial blood pressure by approximately the same magnitude (53.1 ± 7.3 and 55.9 ± 6.1 mmHg respectively). Saline was without effect on arterial blood pressure.

*Portal venous flow (Table III)*

Portal venous flow was significantly increased 10 min following hepatic artery ligation in all 3 groups of rats. Ten minutes following hepatic artery ligation, an infusion of 0.08 $\mu U g^{-1}$ body wt $min^{-1}$ vasopressin, significantly decreased portal venous flow. Conversely, an infusion of 0.16 $\mu U g^{-1}$ body wt $min^{-1}$ vasopressin, significantly increased portal venous flow. An infusion of saline had no effect on portal flow following hepatic artery ligation.

*Liver blood flow (Table IV)*

Hepatic artery ligation had no significant effect on blood flow to normal liver tissue in any of the 3 groups of rats studied. Blood flow to metastatic tissue was however, significantly reduced by hepatic artery ligation in all 3 groups of animals. Ten minutes following hepatic artery ligation an infusion of 0.08 $\mu U g^{-1}$ body wt $min^{-1}$ vasopressin significantly reduced blood flow to normal liver tissue but was without significant effect on blood flow to metastatic tissue. An infusion of 0.16 $\mu U g^{-1}$ body wt $min^{-1}$ vasopressin following hepatic artery ligation increased blood flow to normal tissue but was without effect on the blood flow to metastases. Saline had no effect on blood flow to normal or metastatic liver tissue.

**Table III** The effect of vasopressin on portal venous flow following hepatic artery ligation.

| | Portal venous flow (ml $min^{-1}$) | | |
|---|---|---|---|
| *Rate of infusion of vasopressin ($\mu U$ g body wt $min^{-1}$)* | *Basal* | *10 min after hepatic artery ligation* | *End of vasopressin infusion* |
| 0.08 | 28.7 ± 2.7 | 37.2 ± 2.2[a] | 22.8 ± 1.9[a] |
| 0.16 | 27.1 ± 2.4 | 39.3 ± 2.1[a] | 47.7 ± 2.6[a] |
| Saline | 25.8 ± 2.9 | 37.5 ± 3.2[a] | 38.2 ± 2.5 |

The results are expressed as Mean ± s.e.
[a]Denotes a significant difference from the balsal levels ($P < 0.05$).

**Table IV** The effect of vasopressin on blood flow to normal and metastatic liver tissue following hepatic artery ligation.

| | Blood flow (ml $min^{-1}$ 100 $g^{-1}$) | | | | | |
|---|---|---|---|---|---|---|
| | Normal liver tissue | | | Metastases | | |
| *Rate of infusion of vasopressin ($\mu U g^{-1}$ body wt $min^{-1}$)* | *Basal* | *10 min after ligation* | *End of infusion* | *Basal* | *10 min after ligation* | *End of vasopressin infusion* |
| 0.08 | 45.9 ± 2.3 | 45.2 ± 2.2 | 17.6 ± 2.0[a] | 25.0 ± 2.2 | 8.1 ± 0.9[a] | 7.5 ± 0.7 |
| 0.16 | 44.4 ± 2.9 | 44.9 ± 3.3 | 57.5 ± 3.8[a] | 27.3 ± 4.1 | 8.3 ± 1.2[a] | 8.6 ± 0.9 |
| Saline | 42.3 ± 3.9 | 41.9 ± 3.6 | 43.1 ± 2.6 | 24.6 ± 1.8 | 8.8 ± 1.5[a] | 8.1 ± 1.0 |

The results are expressed as Mean ± s.e.
[a]Denotes a significant difference from the basal levels ($P < 0.05$).

### Discussion

All rats treated with azoxymethane developed some hepatocellular damage but there was no histological evidence of either cirrhosis or foci of microcancer. Furthermore, liver blood flow measured by the clearance of xenon-133 is lower in azoxymethane treated rats than in untreated rats (Mooney & Taylor, 1981). However, hepatic artery ligation has no effect on blood flow to the liver of azoxymethane treated rats (Mooney & Taylor, 1981). Therefore, in this study non-metastatic tissue is referred to as "normal", although we accept that there is some hepatocellular damage and impairment of blood flow.

The results of this study clearly indicate that the effects of vasopressin on portal venous flow and blood flow to normal and metastatic tissue are dependent on the rate of its infusion. The response is biphasic, and accords with previous observations (Mooney *et al.,* 1980). Thus at the lower rate of vasopressin infusion ($0.08\,\mu U\,g^{-1}$ body $wt\,min^{-1}$) portal venous flow was decreased but total blood flow to normal and metastatic liver tissue was increased; at a higher rate of infusion ($0.16\,\mu U\,g^{-1}$ body $wt\,min^{-1}$) these effects were reversed. These contrasting responses were consistently observed (Figure 1).

The precise mechanism whereby vasopressin brings about the observed changes in portal venous flow is not clear. The most generally accepted explanation is that the fall in portal venous flow following vasopressin infusion is the result of constriction of the splanchnic arterioles (Schwartz, 1970), although a direct effect on the intraheptic portal resistance sites has also been suggested (Richardson & Withrington, 1981). In the present study, an infusion of vasopressin resulted in a marked increase in arterial blood pressure, suggesting that splanchnic vasoconstricution was, at least in part, responsible for the observed decrease in portal venous flow. However, it seems unlikely that the increase in portal venous flow following the infusion of $0.16\,\mu U\,g^{-1}$ body $wt\,min^{-1}$ can be explained in terms of changes in splanchnic vasoconstriction since aterial blood pressure was also markedly elevated at this rate of infusion. Possibly, this phenomenon may be caused by a change in the intrahepatic vascular resistances mediated directly or indirectly by high rates of infusion of vasopressin.

Blood flow to normal and metastatic tissue was significantly increased following an infusion of $0.08\,\mu U\,g^{-1}$ body $wt\,min^{-1}$ of vasopressin. Since the blood supply of the liver is derived from the hepatic artery and portal vein and because an infusion of $0.08\,\mu U\,g^{-1}$ body $wt\,min^{-1}$ of vasopressin reduced portal venous flow, the increase in blood flow to both normal and metastatic tissue is probably effected by an increase in hepatic artery flow. This suggestion is supported in the present study by the observation that hepatic artery ligation abolished the increase in blood flow to both normal and metastatic liver tissue following vasopressin infusion while ligation prior to the infusion prevented the increase.

The precise mechanism whereby vasopressin elicits an increased flow in the hepatic artery is not clear. Conn (1973) has suggested that the increase in hepatic artery flow following vasopressin infusion is secondary to a reduction in portal flow. However, the hepatic artery-response to vasopressin is much greater when the hormone is administered intra-arterially than intravenously suggesting a direct effect on the artery (Richardson & Withrington, 1975). Nevertheless, the "dilator" effect of vasopressin on the hepatic arterial bed may be indirect and reflect passive dilation of the pre-arteriolar vessels due to increases in systemic blood pressure (Richardson & Withrington, 1981).

Figure 1

The reduction in blood flow to normal and metastatic liver tissue following a higher rate of vasopressin infusion ($0.16 \mu U g^{-1}$ body wt $min^{-1}$) would again seem to reflect an alteration in hepatic artery flow, since portal venous flow at this rate of infusion was increased. This suggestion is supported by the observation that hepatic artery ligation significantly reduced blood flow to metastatic tissue whose blood supply is derived predominantly from the hepatic artery. Conversely hepatic artery ligation had little effect on blood flow to "normal" liver tissue which receives a blood supply from both the hepatic artery and portal vein, portal venous flow being increased following an infusion of $0.16 \mu U g^{-1}$ body wt $min^{-1}$ vasopressin. Furthermore, ligation of the hepatic artery prior to the infusion of $0.16 \mu U g^{-1}$ body wt $min^{-1}$ vasopressin, abolished the reduction in blood supply to liver metastases when the hormone was infused at this higher rate without prior ligation. Vasopressin has been reported to elicit hepatic artery vasoconstriction in the dog (Richardson & Withrington, 1975). Possibly, therefore, at a rate of infusion of $0.16 \mu U g^{-1}$ body wt $min^{-1}$ the hepatic artery cannot escape the generalised vasoconstrictor properties of the hormone, and this results in a decreased flow through the hepatic artery and an overall decrease in liver blood flow.

The observations of this study on the effects of different rates of infusion of vasopressin on hepatic haemodynamics may have important practical implications in the management of patients presenting with liver metastases. Firstly, vasopressin infusion at a rate comparable to that currently used for the management of bleeding oesophageal varices, may, by reducing portal venous flow, potentiate the effects of hepatic artery ligation or embolisation in rendering tumour tissue ischaemic. Secondly, since this rate of vasopressin infusion increases hepatic artery flow, the simultaneous administration of vasopressin and a cytotoxic agent may ensure a preferential delivery of the latter to liver metastases. Lastly, a higher rate of infusion of vasopressin that is currently used for the emergency control of bleeding oesophageal varices, by increasing portal venous flow, may potentiate cytotoxic therapy given via the portal vein in combination with hepatic artery ligation or embolisation.

### References

ACKERMAN, N., LIEN, W.M., KONDI, E.S. & SILVERMAN, N.A. (1969). The blood supply of experimental liver metastases. 1. The distribution of hepatic artery and portal vein blood to "small" and large tumours. *Surgery*, **66**, 1967.

BENGMARK, S. & HAFSTROM, L. (1969). The natural history of primary and secondary malignant tumours of the liver. *Cancer*, **23**, 198.

BREEDIS, C. & YOUNG, G. (1954). The blood supply of neoplasms in the liver. *Am. J. Pathol.*, **30**, 969.

CHOJKIER, M., GROSZMAN, R.J., ATTERBURY, C.E. & 12 others. (1979). A controlled comparison of continuous intra-arterial and intravenous infusion of vasopressin in haemorrhage from oesophageal varices. *Gastroenterology*, **77**, 540.

CONN, H. Jr. (1961). Equilibrium distribution of radioxenon in tissue: Xenon haemoglobin association curve. *J. Applied Physiol.*, **16**, 1065.

CONN, H.O. (1973). Hepatic arterial escape from vasopressin induced vasoconstriction: An angiographic investigation. *Am. J. Roentol. Theoret. Nucl. Med.*, **1**, 102.

GELIN, L.E., LEWIS, D.H. & NILSSON, C. (1968). Liver blood flow in man during abdominal. surgery. *Acta Hepato-splenologica* (Stuttgart) **15**, 13.

GRAGE, T.B., VASSILOPOULOS, P.P., SHINGLETON, W.W. *et al.* (1979). Results of prospective randomised study of hepatic artery infusion with 5-fluorouracil versus intravenous 5-fluorouracil in patients with hepatic metastases from colorectal cancer. A Central Oncology Group Study. *Surgery*, **86**, 550.

KEHNE, J.H., HUGHES, F.A. & GOMPERTZ, M.L. (1956). The use of surgical pituitrin in the control of oesophageal varices: An experimental study and report of two cases. *Surgery*, **39**, 917.

LEWIS, D.H. (1970). Intra-operative blood flow measurements with Xenon washout technique. *Prog. Surg.*, **8**, 74.

MATTSON, W., JOHNSON, S., HELLENKANT, C. & HALLSTEN, L. (1980). Short-term intra-arterial mitomycin in hepatic metastases. *Acta Radiol.*, **19**, 321.

McKENZIE, R.J., LEIBERMAN, D.P., MATHIE, R.T., RICE, G.C., HARPER, A.M. & BLUMGART, L.H. (1976). Liver blood flow measurement. The interpretation of Xenon-133 clearance curves. *Acta. Chir. Scand.*, **142**, 519.

MOONEY, B. (1981). *Colorectal liver metastases – a multidisciplinary study*. Submitted for the degree of M.Ch., University College Dublin.

MOONEY, B. & TAYLOR, I. (1981). The effect of hepatic arterial ligation on spontaneously developing colorectal metastases in the rat. *Clin. Oncol.*, **8**, 231.

MOONEY, B., TAYLOR, A., SHIELDS, R. & TAYLOR, I. (1980). Vasopressin and liver blood flow – an experimental dose response study. *Br. J. Surg.*, **67**, 831.

NAMAMURA, K. *et al.* (1981). A non-resectable hepatoma after hepatic artery ligation combined with infusion chemotherapy; an eight year survival. *Jap. J. Surg.*, **11**, 80.

NIELSON, J., BALSLEV, I., FENGER, H.J., JENSEN, H.E. & KRAGELUND, E. (1973). Carcinoma of the rectum with liver metastases. Prognosis and operative indication. *Acta Chir. Scand.*, **139**, 479.

OXLEY, E.M. & ELLIS, H. (1969). Prognosis of carcinoma of the large bowel in the presence of liver metastases. *Br. J. Surg.*, **56**, 149.

REED, M.L., VIATKEVICIUS, V.K., AL-SARRAF, M. & 6 others. (1981). The practicability of chronic hepatic artery infusion therapy of primary and metastatic hepatic malignancies: Ten year results of 124 patients in prospective protocol. *Cancer,* **47,** 402.

RICHARDSON, P.D.I. & WITHRINGTON, P.G. (1975). The effects of intra-aterial and intra-portal injections of vasopressin on the simultaneously perfused hepatic arterial and portal venous vascular beds of the dog. *Cir. Res.,* **43,** 496.

RICHARDSON, P.D.I. & WITHRINGTON, P.G. (1981). Liver blood flow effects of drugs and hormones in liver blood flow. *Gastroenterology,* **81,** 356.

SCHWARTZ, S.I. (1970). Influence of vasoactive drugs on portal circulation. *Ann. Acad. Sci.,* **170,** 296.

SHIELDS, R. (1977). The management of portal hypertension and bleeding oesophageal varices. *Br. J. Hosp. Med.,* **17,** 126.

TAYLOR, I., BENNETT, R. & SHERRIFF, S. (1979). The blood supply of colorectal liver metastases. *Br. J. Cancer.,* **39,** 749.